# PReS-FINAL-2143: Treat-to-target strategy in juvenile idiopathic arthritis: experience in 175 newly-diagnosed patients

**DOI:** 10.1186/1546-0096-11-S2-P155

**Published:** 2013-12-05

**Authors:** G Negro, A Consolaro, S Verazza, G Bracciolini, S Rosina, R Pignataro, A Civino, A Martini, A Ravelli

**Affiliations:** 1 IRCCS G.Gaslini, Genova, Italy; 2 Azienda Ospedaliera Card. Panico, Tricase, Italy

## Introduction

The recent advances in the management of juvenile idiopathic arthritis (JIA) have increased considerably the potential to achieve disease remission or, at least, low levels of disease activity, and have consequently moved the therapeutic aims towards the attainment of inactive disease (ID). Complete disease quiescence is regarded as the ideal therapeutic target because its achievement helps preventing further joint damage and disability and may enhance physical function and quality of life.

## Objectives

These issues have led to suggest that a tight control approach should be adopted in the management of JIA. We describe our experience with treat-to-target strategy.

## Methods

Starting in March 2007, a treat-to-target approach to the management of all children with JIA first seen in the senior author's clinic was implemented, setting achievement of ID as primary goal and of minimal disease activity (MDA) or parent-acceptable symptom state (PASS) as secondary goals. In case primary goal was not reached, treatment was intensified as deemed necessary. Patient records were reviewed to evaluate the frequency of achievement of the therapeutic goals at 6, 12, 18 and 24 months following initial evaluation. ID, MDA and PASS were defined according to both established criteria and Juvenile Arthritis Disease Activity Score (JADAS) cutoffs. The outcome of patients who achieved or did not achieve ID at last follow-up visit was compared by means of the Juvenile Arthritis Functionality Scale (JAFS) and the Pediatric Rheumatology Quality of Life scale (PRQL).

## Results

A total of 175 patients (77.7% females) were enrolled. The most common ILAR subtypes were persistent oligoarthritis (53.1%), extended oligoarthritis (14.9%), and RF-negative polyarthritis (14.3%); 3.4% of patients had systemic arthritis. The median age at disease onset was 2.8 years. At baseline visit, the median age was 3.5 years and the median disease duration was 0.2 years. Initial therapeutic interventions included intra-articular corticosteroid injection (84%), methotrexate (28%), systemic corticosteroids (5.7%), and biologic medications (1.1%). The frequency of achievement of treatment goals at study endpoints is shown in the table. At last follow-up visit, patients who had achieved ID had better functional ability (p = 0.007) and physical well-being (p = 0.007) than those who did not. The frequency of clinical remission on medication was 29.2%. (Table 1)

**Table 1:** 

	Inactive disease (ID)	Minimal disease activity (MDA)	Parent-acceptable symptom state (PASS)	JADAS10 ≤ 1 (ID)	JADAS10 ≤ 2/3.8 (MDA)	JADAS10 ≤ 3.5/5.4 (PASS)
**6 months** **N(%)**	50(35.2)	62(43.7)	76(66.1)	43(31.6)	43(31.6)	64(47.1)
**12 months** **N(%)**	48(41)	71(60.7)	77(74.8)	46(41.4)	58(52.3)	66(59.5)
**18 months** **N(%)**	46(47.4)	60(61.9)	69(82.1)	43(46.2)	60(64.5)	67(72)
**24 months** **N(%)**	45(50.6)	5(59.6)	53(70.7)	33(39.3)	40(47.6)	52(61.9)

## Conclusion

At 2 years after initial visit, a substantial percentage of patients had reached the states of ID or MDA or were in PASS. Patients who achieved ID had better physical function and well-being than those who did not. These findings suggest that the implementation of a treat-to-target approach may help improve patient outcomes.

## Disclosure of interest

None declared.

